# Elucidating therapeutic molecular targets in premenopausal Asian women with recurrent breast cancers

**DOI:** 10.1038/s41523-018-0070-x

**Published:** 2018-07-26

**Authors:** Yoon-Sim Yap, Angad P. Singh, John H. C. Lim, Jin-Hee Ahn, Kyung-Hae Jung, Jeongeun Kim, Rebecca A. Dent, Raymond C. H. Ng, Sung-Bae Kim, Derek Y. Chiang

**Affiliations:** 1 Division of Medical Oncology, National Cancer Centre, Singapore, Singapore; 2 Faculty of Health Sciences, School of Medicine, University of Adelaide, Adelaide, Australia; 3 Oncology Next-Generation Diagnostics, Novartis Institutes for Biomedical Research, Cambridge, USA; 4 Department of Clinical Trials and Epidemiological Sciences, National Cancer Centre, Singapore, Singapore; 5 Department of Oncology, Asan Medical Center, University of Ulsan College of Medicine, Seoul, Republic of Korea

## Abstract

Breast cancer is an increasing problem in Asia, with a higher proportion of premenopausal patients who are at higher risk of recurrence. Targeted sequencing was performed on DNA extracted from primary tumor specimens of 63 premenopausal Asian patients who relapsed after initial diagnosis of non-metastatic breast cancer. The most prevalent alterations included: *TP53* (65%); *PIK3CA* (32%); *GATA3* (29%); *ERBB2* (27%); *MYC* (25%); *KMT2C* (21%); *MCL1* (17%); *PRKDC, TPR, BRIP1* (14%); *MDM4, PCDH15, PRKAR1A, CDKN1B* (13%); *CCND1, KMT2D, STK11*, and *MLH1* (11%). Sixty of the 63 patients (95%) had at least one genetic alteration in a signaling pathway related to cell cycle or p53 signaling. The presence of *MCL1* amplification, HIF-1-alpha transcription factor network pathway alterations, and direct p53 effectors pathway alterations were independent predictors of inferior overall survival from initial diagnosis. Comparison with non-Asian premenopausal tumors in The Cancer Genome Atlas (TCGA) revealed a higher prevalence of *TP53* mutations among HER2-positive cancers, and more frequent *TP53, TET2*, and *CDK12* mutations among hormone receptor-positive HER2-negative cancers in our cohort. Given the limited number of non-Asian premenopausal breast cancers that had relapsed in TCGA, we compared the frequency of mutations in our cohort with 43 premenopausal specimens from both TCGA and International Cancer Genome Consortium that had relapsed. There was a trend toward higher prevalence of *TP53* mutations in our cohort. Certain genomic aberrations may be enriched in tumors of poor-prognosis premenopausal Asian breast cancers. The development of novel therapies targeting these aberrations merit further research.

## Ethnic diversity: distinct molecular profiles in tumors from premenopausal Asian women

Younger women in Asia with recurrent breast cancer seem to have a higher rate of mutations in the tumor suppressor gene *TP53* than do women elsewhere—a finding that could guide drug development in Asia. Yoon-Sim Yap from the  National Cancer Centre Singapore and coworkers sequenced DNA extracted from the tumor samples of 63 premenopausal women from Singapore and South Korea who relapsed following treatment for non-metastatic breast cancer. The researchers analyzed hundreds of cancer-related genes, and found that the vast majority of women harbored mutations in at least one gene linked to regulating the cell cycle of *TP53* signaling. The prevalence of mutations in certain genes, including *TP53* itself, was higher than observed previously in non-Asian cohorts, highlighting the need to consider ethnic diversity in genomic studies of breast cancer and in drug development.

## Introduction

Breast cancer is an increasing health problem in East Asia where the incidence of breast cancer has been rising dramatically over the past few decades. For the younger age groups, the incidence of breast cancer in several Asian countries has even surpassed that in the United States.^1^

This phenomenal increase in breast cancer rates cannot be solely attributed to the effects of screening and improved data capture. The adoption of a westernized lifestyle in recent generations has been suggested as a major cause of this trend. Hormonal risk factors such as earlier age at menarche, low parity, delayed age at first birth, rising body mass index, and dietary factors with increased consumption of fat and animal-source products, have been implicated.^1–3^ In addition, the higher exposure to environmental pollutants with estrogenic effects among East Asian women may also contribute to the increasing incidence.^3^

There are distinct ethnic differences in the biology of certain cancers such as lung adenocarcinoma, where the frequency of mutations in *EGFR* (epidermal growth factor receptor) is significantly higher in East Asians.^4^ To date, such differences have not been reported in breast cancer. However, the age of onset is generally younger in East Asia.^1,3,5^ Over 40% of breast cancers in Asia are diagnosed in women under 50 years of age, compared with ~ 20% in western countries.^5^ Younger women with early breast cancer are at higher risk of relapse and death from breast cancer; this may be related to differences in tumor and/or host biology.^6^ Comparison of breast cancers from young and elderly women in The Cancer Genome Atlas (TCGA) revealed an association of *GATA3* mutations and chr6q27 deletions with younger age, and higher expression of gene signatures related to proliferation, stem cell features, and endocrine resistance.^7^

Given the limited data on Asian breast cancers, and the higher prevalence of premenopausal patients, it is critical to elucidate the genomic landscape in premenopausal Asian patients who relapse after initial diagnosis of non-metastatic breast cancer. The main objective of this study is to identify actionable genomic aberrations in the primary breast cancers from premenopausal Asian patients who subsequently relapse. We also aimed to identify the genomic aberrations associated with inferior survival outcomes, and to compare the frequency of these genomic aberrations with non-Asian premenopausal tumors in publicly available databases such as TCGA and International Cancer Genome Consortium (ICGC).

## Results

### Clinical and pathological characteristics

A total of 110 patients were identified over the study period from September 2014 to May 2016 to have relapsed from breast cancer and had been premenopausal at initial diagnosis. DNA was extracted from formalin-fixed paraffin-embedded (FFPE) sections of the initial breast cancer primary from these patients, of which 63 unique samples passed quality control. Hence, the patient population with sequencing data consists of 63 women who were premenopausal at initial diagnosis from 2008 to 2015 (Table 1). The median age at diagnosis was 42 years (range 25–49 years). Majority of the cases were stage 2 (41.3%) or stage 3 (52.4%) at diagnosis. The most common immunohistochemical subtype was “luminal” (defined as estrogen receptor (ER)-positive and/or progesterone receptor (PR)-positive, as well as human epidermal growth factor receptor 2 (HER2)-negative in this study) at 42.9%, followed by HER2-overexpressing subtype (regardless of hormone receptor status) at 33.3%, whereas triple-negative cases comprised 23.8% of patients in the cohort. The median time to relapse was 23 months (range 6–150 months). The median overall survival (OS) was 59 months (95% confidence interval (CI) 45–79 months) after initial diagnosis, and 25 months (95% CI 20–27 months) after relapse.

**Table 1:** Patient and primary tumor characteristics

	Age at diagnosis (years)			Total (*n* = 63)
Characteristics	< 35 (*n* = 16) no. (%)	35–44 (*n* = 28) no. (%)	≥ 45 (*n* = 19) no. (%)	No. (%)
Age in years, median (range)		42 (25–49)		
Follow-up in months, median (range)		44 (8–168)		
Ethnicity				
Chinese	6 (37.5)	13 (46.4)	9 (47.4)	28 (44.4)
Korean	7 (43.8)	10 (35.7)	5 (26.3)	22 (34.9)
Malay	0	3 (10.7)	5 (26.3)	8 (12.7)
Indian	2 (12.5)	0	0	2 (3.2)
Others	1 (6.3)	2 (7.1)	0	3 (4.8)
AJCC stage				
0	0	0	1 (5.3)	1 (1.6)
I	0	1 (3.6)	1 (5.3)	2 (3.2)
II	5 (31.3)	11 (39.3)	10 (52.6)	26 (41.3)
III	10 (62.5)	16 (57.1)	7 (36.8)	33 (52.4)
Unknown (TxN1M0)	1 (6.3)	0	0	1 (1.6)
Grade				
1	0	0	0	0
2	6 (37.5)	11 (39.3)	6 (31.6)	23 (36.5)
3	10 (62.5)	16 (57.1)	13 (68.4)	39 (61.9)
Unknown	0	1 (3.6)	0	1 (1.6)
ER status at diagnosis				
Positive	9 (56.3)	19 (67.9)	8 (42.1)	36 (57.1)
Negative	7 (43.8)	9 (32.1)	11 (57.9)	27 (42.9)
PR status at diagnosis				
Positive	7 (43.8)	13 (46.4)	7 (36.8)	27 (42.9)
Negative	9 (56.3)	15 (53.6)	12 (63.2)	36 (57.1)
HER2 status at diagnosis				
Positive	4 (25.0)	11 (39.3)	6 (31.6)	21 (33.3)
Negative	12 (75.0)	17 (60.7)	13 (68.4)	42 (66.7)
Immunohistochemical subtype				
“Luminal” (ER and/or PR + , HER2−)	7 (43.8)	13 (46.4)	7 (36.8)	27 (42.9)
HER2-overexpressing (regardless of ER/PR)	4 (25.0)	11 (39.3)	6 (31.6)	21 (33.4)
Triple-negative (ER−, PR−, HER2−)	5 (31.3)	4 (14.3)	6 (31.6)	15 (23.8)
Histology				
Ductal	15 (93.8)	25 (89.3)	15 (78.9)	55 (87.3)
Lobular	0	0	2 (10.5)	2 (3.2)
Others	1 (6.3)	3 (10.8)	2 (10.6)	6 (9.6)
Adjuvant/neoadjuvant chemotherapy				
No	0	3 (10.7)	4 (21.1)	7 (11.1)
Yes	16 (100)	25 (89.3)	15 (78.9)	56 (88.9)
Adjuvant/neoadjuvant anti-HER2 therapy (among HER2 +)				
No	0	1 (9.1)	1 (16.7)	2 (9.5)
Yes	4 (100)	10 (90.9)	5 (83.3)	19 (90.5)
Adjuvant/neoadjuvant endocrine therapy (among ER + and/or PR +)				
No	0	3 (15.8)	2 (22.2)	5 (13.5)
Yes	9 (100)	16 (84.2)	7 (77.8)	32 (86.5)

### Prevalence of genomic alterations

**Fig. 1:**
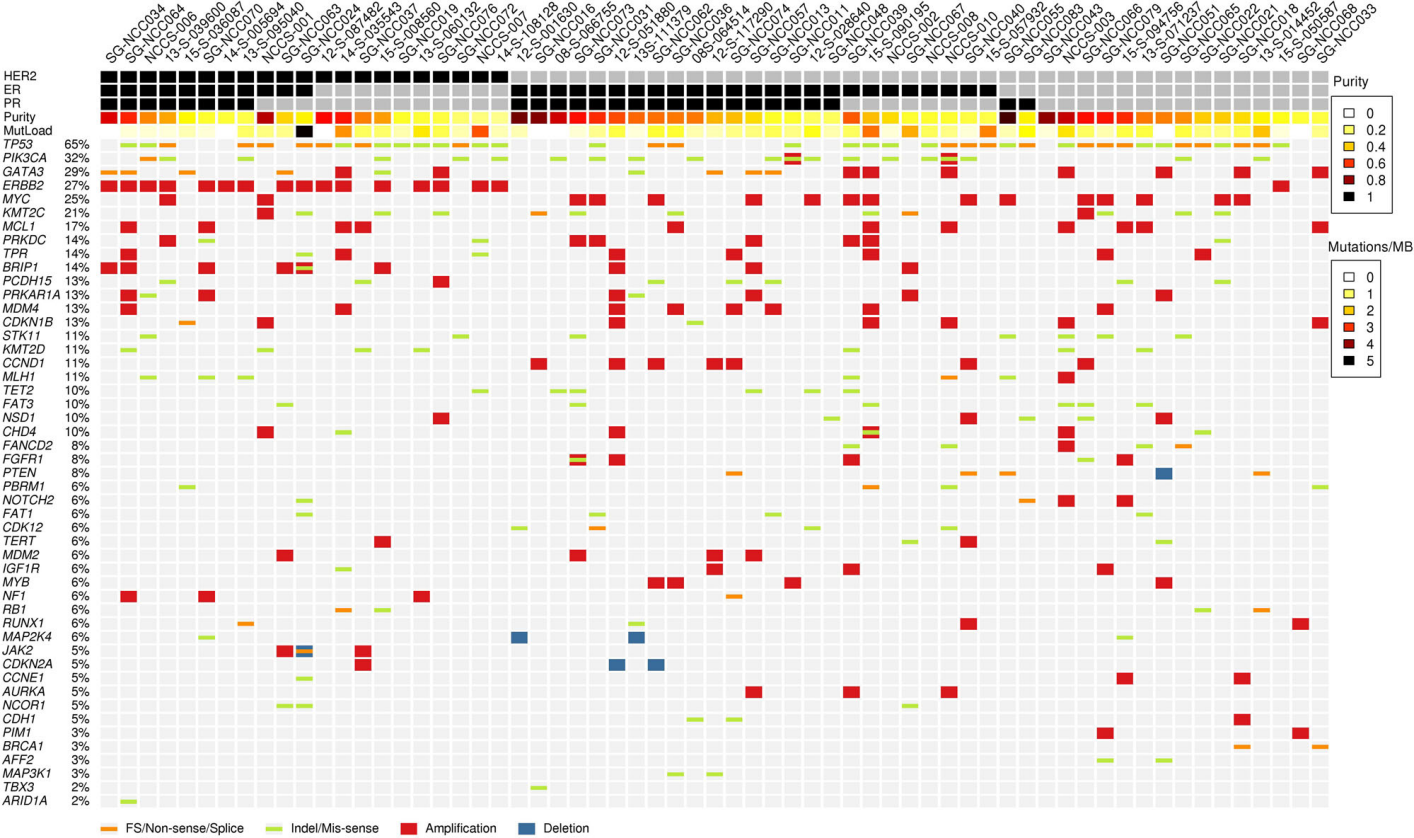
Genomic profile of 63 tumor samples ordered by receptor status: HER2 + (*n* = 21), hormone receptor (HR) + and HER2 − (*n* = 27), and triple-negative (*n* = 15), and purity. The figure lists the prevalence of mutations, indels, and copy number variations for recurrently mutated genes in breast cancer and the mutation load of each sample. Purity and mutation load are indicated for each sample. The darker the shade, the higher the value

Among the 63 cases with sequencing data, a total of 406 single nucleotide variants (SNVs) and 52 indels were found (Fig. 1, Supplementary Table S1). The most prevalent mutations and amplifications in oncogenes included: *PIK3CA* (32%); *GATA3* (29%); *ERBB2* (27%); *MYC* (25%); *MCL1* (17%); *PRKDC* (14%); *MDM4* (13%); and *CCND1* (11%). The most prevalent alterations in tumor suppressor genes included: *TP53* (65%); *KMT2C* (21%); *PCDH15* (13%); *KMT2D* (11%); *STK11* (11%); and *MLH1* (11%). Sixty of the 63 patients (95%) had at least one genetic alteration in a signaling pathway related to cell cycle or p53 signaling (Supplementary Table S2).

### Association between clinical, molecular characteristics, and survival outcomes

From multivariable analysis where the resultant model incorporated age, immunohistochemical subtype as well as other clinicopathologic characteristics including the most common gene alterations, age (hazards ratio (HR) 1.10 per year increase; 95% CI 1.03–1.17; *p* = 0.006) and presence of *MCL1* amplification (HR 4.24; 95% CI 1.62–11.07; *p* = 0.003) were found to be independent predictors of overall survival (OS) from initial diagnosis (Supplementary Table S3).

Given that mutations in different genes can compromise a particular pathway and this may provide a better prediction of outcomes compared with alterations in individual genes, we also tested for associations between pathway alterations and OS (Supplementary Table S4). From multivariable analysis, only age (HR 1.09 per year increase; 95% CI 1.02–1.17; *p* = 0.008), presence of alterations in the HIF-1-alpha transcription factor network pathway, which includes *MCL1* among other genes (HR 2.57; 95% CI 1.18–5.60; *p* = 0.017), and presence of alterations in direct p53 effectors (HR 3.61; 95% CI 1.08–12.01; *p* = 0.036) were predictive of inferior OS from initial diagnosis.

### Comparison with premenopausal non-Asian breast cancers in TCGA

**Fig. 2:**
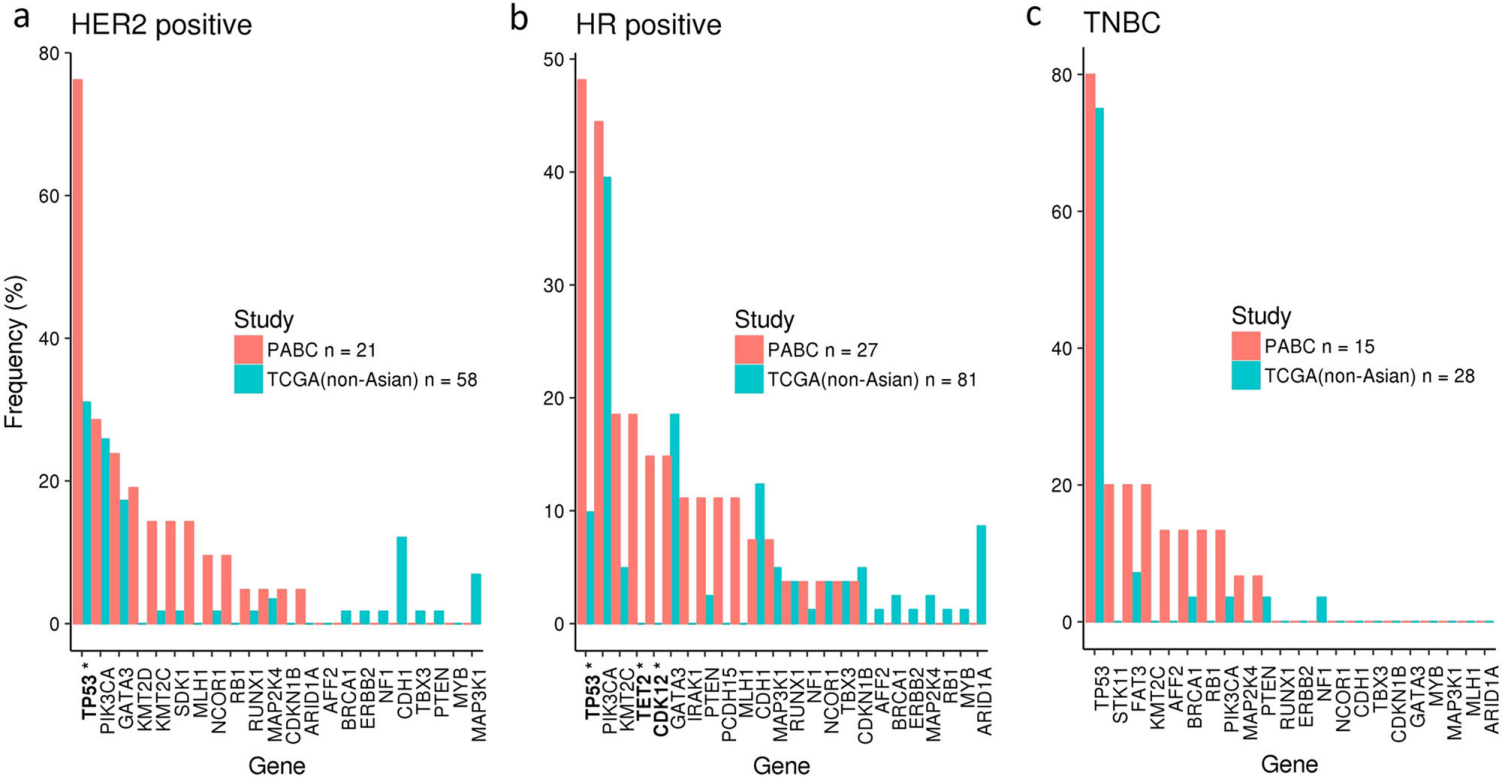
Mutation prevalence in our cohort (PABC: Premenopausal Asian Breast Cancers) and comparison with premenopausal, non-Asian breast cancer patients in The Cancer Genome Atlas. Starred genes indicate significant difference from TCGA data (FDR *q* < 0.05). **a** Comparison of 21 HER2-positive samples against 58 non-Asian TCGA samples. **b** Comparison of 27 hormone receptor-positive samples against 81 non-Asian TCGA samples. **c** Comparison of 15 triple-negative breast cancers against 28 non-Asian TCGA samples

The prevalence of mutations was compared between this cohort and 167 premenopausal, non-Asian breast cancer patients from TCGA^8^ with information on hormone receptor and HER2 status (Fig. 2). The small number of Asian premenopausal cases in TCGA cohort, with nine hormone receptor-positive, HER2-negative tumors, five HER2-positive tumors, and no triple-negative cancers, precludes any meaningful comparison with our cohort. Given the variability in copy number calling on a number of platforms with varying sample purity levels across different laboratories in TCGA, it is challenging to perform an accurate comparison of copy number alterations with our data set. Hence, comparisons with genomic data were made only for the mutations.

Comparison of the HER2-positive cases (regardless of hormone receptor status) revealed a significantly higher prevalence of *TP53* (76%) mutations (FDR *q* < 0.05) in our cohort (Fig. 2a). Among the hormone receptor (HR)-positive and HER2-negative cases, there was a significantly higher prevalence of *TP53* (48%), *TET2* (18%), and *CDK12* (15%) mutations (FDR *q* < 0.05) (Fig. 2b) than TCGA cohort. With the small number of triple-negative breast cancer patients, no significant difference in genetic alterations was found (Fig. 2c).

### Comparison with premenopausal breast cancers in TCGA and ICGC that subsequently relapsed

**Fig. 3:**
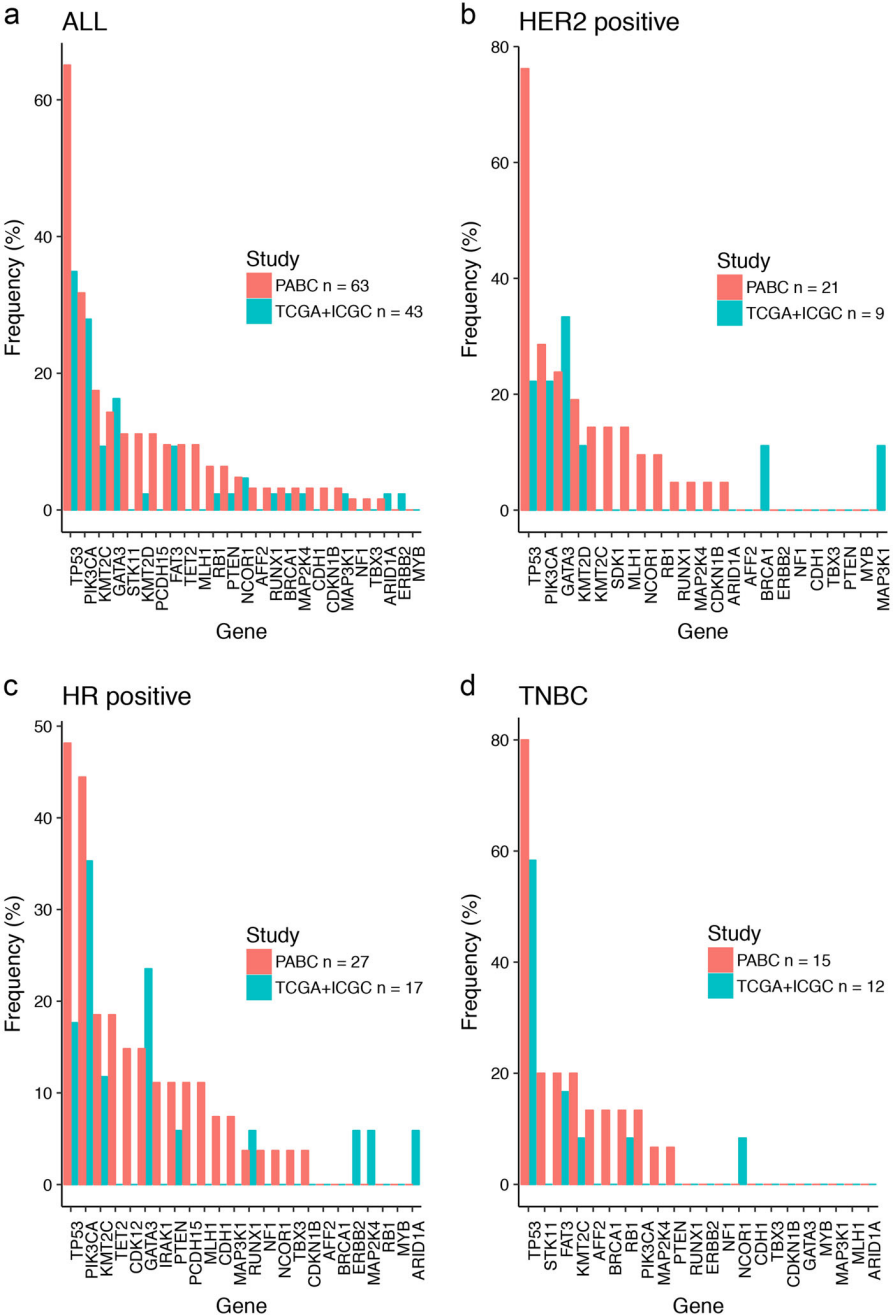
Mutation prevalence in our cohort (PABC: Premenopausal Asian Breast Cancers) and comparison with 43 premenopausal breast cancer patients who had relapsed from both TCGA and ICGC studies. **a** Comparison of all 63 samples in the cohort against 43 premenopausal breast cancer patients (all subtypes) who had relapsed from both TCGA and ICGC studies (25 non-Asian premenopausal from TCGA and 18 premenopausal from ICGC-EU). **b** Comparison of 21 HER2-positive samples against nine TCGA + ICGC samples. **c** Comparison of 27 hormone receptor-positive samples against 17 TCGA + ICGC samples. **d** Comparison of 15 triple-negative breast cancers against 12 TCGA + ICGC samples

To compare frequently mutated genes between Asian and non-Asian premenopausal breast cancers, we surveyed 43 premenopausal breast cancer patients who had relapsed from both TCGA and ICGC studies (25 non-Asian premenopausal from TCGA and 18 premenopausal from ICGC-EU) (Fig. 3).^8,9^ ICGC is currently the only other publicly available data set with information on recurrence status. Comparison between all patients revealed a higher prevalence of *TP53* mutations (65% versus 35%) in our cohort (Fig. 3a). However, the limited number of cases in both cohorts did not result in statistical significance.

## Discussion

Although large-scale sequencing projects have unraveled the complex architecture of breast cancers in the West,^8–11^ data on the genomic landscape of Asian breast cancers, including premenopausal tumors, remains limited.^12,13^ Given that studies on next-generation sequencing of Asian breast cancers that are currently published focus on special subsets, it is not possible to compare the mutation frequency from our cohort with an Asian premenopausal breast cancer cohort that has not relapsed. For example, in the study by Kim et al.^12^ from Korea, whole-exome sequencing was performed on 34 metastatic breast cancer specimens, with patient age ranging from 26.5 to 75.7 years. In another study by Lee et al.,^13^ also from Korea, a total of 78 normal-paired breast cancers were subjected to whole exome and RNA sequencing, but only 35 were from patients under the age of 50 years. As illustrated in Figs. 2 and 3, the numbers of premenopausal non-Asian tumors profiled in large sequencing projects such as TCGA and ICGC are also limited. In this study, we have focused on characterizing the genomic profile of premenopausal Asian breast cancers that ultimately relapse, given that this is an area of unmet need. Similar to reports from other sequencing studies,^8–10^ there is a large number of genes where the frequency of alterations is < 10%, reflecting the heterogeneity of the genomic landscape in breast cancer. Each tumor was also distinct with its own unique set of alterations.

The prevalence of *TP53* mutations is higher among the hormone receptor-positive and HER2-positive subtypes in our cohort compared to non-Asian premenopausal cancers in TCGA, along with elevated frequencies of *TET2* and *CDK12* mutations among hormone receptor-positive patients. The finding that *TP53* is more frequently mutated overall in our series may be related to the pre-selection of poor-prognosis tumors. However, this is also consistent with the findings from a recent study on unselected breast cancers from Chinese women who reported a lower prevalence of PAM50 luminal A subtype and a higher prevalence of luminal B subtype compared to Caucasian series.^14^ The frequency of HER2-overexpressing and basal-like breast cancers was similar between Chinese and Caucasian breast cancers. Luminal B subtypes are associated with more aggressive biology and higher risk of relapse, and often harbor *TP53* mutations that have been implicated in resistance to endocrine and cytotoxic therapies.^8,10,15,16^ In addition, the frequency of *TP53* mutations appears higher in our cohort compared with non-Asian premenopausal tumors in TCGA and ICGC that had relapsed, though this finding will require further validation in a larger study. Although targeting the loss of function of tumor suppressor genes remains a major challenge, there may be opportunities in the near future with the advent of immune-based and epigenetic strategies, as well as compounds targeting downstream effectors in the affected pathways.

Other alterations present at higher frequencies in our series of poor-prognosis tumors include *CDK12* and *TET2*. Cyclin-dependent kinase 12 (CDK12) is a regulatory kinase, which protects cells from genomic instability. Recurrent *CDK12* mutations in breast and ovarian cancers are associated with defects in DNA repair.^17^ Silencing of *CDK12* has been shown to activate the mitogen-activated protein kinase signaling pathway, leading to endocrine therapy resistance through loss of ER dependence.^18^ TET2 (tet-eleven translocation 2) from the TET family of DNA dioxygenases functions as DNA demethylases, antagonizing DNA methyltransferases-mediated DNA methylation and gene repression. Knockdown of *TET2* in breast cancer cells decreases epithelial cell adhesion molecule and E-cadherin, increasing cell invasiveness.^19^

MCL1 (myeloid cell leukemia-1), a member of the anti-apoptotic pro-survival Bcl-2 family, can be amplified in all subtypes of breast cancer. In our cohort, *MCL1* amplification was predictive of inferior OS, likely related to the resistance to endocrine, cytotoxic and anti-HER2 therapies reported in preclinical studies.^20,21^ The in vitro activity of MCL1 inhibitors alone or in combination with other anti-cancer drugs provide a strong rationale for their clinical development.^22,23^

Limitations of our study include the relatively small sample size, and the limited number of premenopausal breast cancers in TCGA and ICGC databases for comparison. Information on ethnicity was not documented for the ICGC cohort, although most of the patients were non-Asian. The suboptimal quality of DNA from older FFPE specimens also led to greater representation of more recent aggressive cases such as HER2-positive and triple-negative cases, which relapsed soon after initial diagnosis. This bias may partly explain why there were no significant survival differences among the different immunohistochemical subtypes, without association of younger age with worse survival. Conversely, increasing age was associated with worse OS in this selected premenopausal cohort, unlike studies that reported higher risk of relapse among younger women in non-metastatic cancers.^6^ The retrospective nature of our study may also create some bias and heterogeneity, as it was not performed as a prospective cohort study or clinical trial.

Although it is possible to identify increased copy number with the targeted gene panel, the low tumor purity of FFPE samples makes it difficult to distinguish between lack of tumor content and a true copy number loss. Hence, the computational loss of heterozygosity calls in this cohort may not be reliable and are not reported. Germline DNA was not available for this study as a matched control. However, our computational pipelines have been optimized to exclude germline and false positive sites, including for tumor suppressor genes. At last, we did not profile the recurrent specimens to interrogate the genomic evolution in the metastatic process. However, a recent study has demonstrated that new driver mutations private to the metastatic lesions are acquired later in the metastatic lineage.^24^ Hence, the primary tumor genome can still serve as a good proxy for the cells that seed the distant sites, and remains relevant in the development of therapeutic strategies in the adjuvant setting to prevent relapse.

In conclusion, our study has provided insights into the molecular profiles of Asian premenopausal breast cancer associated with relapse. The heterogeneity of breast cancers highlights the need to explore ethnic diversity in the genomic landscape. Standard systemic adjuvant therapies may be ineffective in these patients, and novel approaches exploiting the underlying tumor biology merit further research.

## Methods

### Patients and samples

Women who were premenopausal on initial diagnosis of breast cancer and relapsed subsequently were identified during outpatient clinic visits, inpatient hospital admissions, or from the institutional database at National Cancer Centre Singapore and Asan Medical Centre, South Korea. Demographic data, histopathological features, treatment details and patient outcome (time to relapse and OS) were obtained from medical records. The definitions of ER, PR, HER2 and positivity in this study were based on the latest recommendations by the American Society of Clinical Oncology and the College of American Pathologists.^25,26^ The study was approved by SingHealth Centralised Institutional Review Board and Asan Medical Center Institutional Review Board in the respective institutions. This research was conducted in accordance with all relevant guidelines and procedures, with signed informed consent obtained from patients over 2014–2016, and waiver of consent from deceased patients as granted by the local ethics committees.

### Next-generation sequencing analysis of somatic mutations, indels, and copy number calls

DNA was extracted from FFPE sections of the initial breast cancer primary with at least 50% tumor percentage from 110 patients. DNA libraries were generated using the TruSeq Nano Library Preparation kit (Illumina). Hybridization capture to a customized Agilent SureSelectXT panel was used to enrich coding regions from 567 cancer-related genes. A total of 63 unique samples passed quality control metrics for sequencing on a HiSeq-2500 with average coverage of at least 300X and were included in the analysis. The NGDx PanCancer version 2 panel interrogates the entire coding sequence of 567 cancer-related genes plus select introns from 57 genes often rearranged or altered in solid tumor cancers (Supplementary Table S5).

Sequencing data were processed as follows: sequence reads were aligned with BWA-MEM to the reference human genome (build hg19) (https://arxiv.org/abs/1303.3997). Next, PCR duplicates were marked with Picard (http://broadinstitute.github.io/picard/) and the Genome Analysis ToolKit (GATK) was used for local realignment and base quality score recalibration.^27,28^ Single nucleotide variants (SNVs) were called using MuTect,^29^ indels using Pindel^30^ and copy number was called using PureCN.^31^ SNVs and indels were annotated with dbSNP v146,^32^ COSMIC v70,^33^ and for various other databases using the SnpEff tool.^34^

SNVs and indels were filtered for germline variants and artifacts using a pool of normal control samples and an extensive set of filters using the dbSNP and 1000 genomes databases. SNV were considered as sequencing artifacts if they were observed in at least 2 of 50 normal samples, or occurred within simple repeats or segmental duplications in the UC Santa Cruz reference genome annotation. SNV were considered as germline variants if they matched one of the following three criteria: (1) found in at least two samples in the Exome Sequencing Project database; (2) annotated as common by dbSNP (> 5% minor allele fraction in one or more populations, as determined by the G5 flag in dbSNP); (3) identified in the 1000 Genomes Project. Putative germline variants could be rescued if they were annotated in the COSMIC database. Otherwise, germline variants were excluded from further analyses. Non-silent variants with a minimum of five supporting reads and total read coverage ≥ 50 × were retained. For indels, the minimum threshold was set at four reads. Indel length size was capped at 100 bp.

Allele-specific copy number was obtained from coverage data for probe intervals. Coverage was first normalized for GC-bias. The purity, ploidy, and copy number were jointly estimated for each sample using PureCN.^31^ For coverage normalization, PureCN uses a pool of normal samples to determine a ‘best-match’ set of normals using Principal Component Analysis. Probe-level copy number values are averaged into per-gene values before reporting. We also conducted pathway level analysis of 16 most relevant pathways in cancer development and progression (Supplementary Table S2).

### Genomic analysis of TCGA and ICGC samples

TCGA data were obtained from the cBioPortal and included the most comprehensive set of cases from the “TCGA Provisional” data set (http://www.cbioportal.org/study?id=brca_tcga). The ICGC data included the BRCA-EU study and was obtained from the ICGC Portal (https://dcc.icgc.org/projects/BRCA-EU). Since this was a whole-genome study, we retained mutations in genes from the 567 cancer-related genes in our PanCancer panel. All intergenic, intronic, upstream, downstream, and other silent mutations or others of no significance were discarded for comparison purposes. In addition, single nucleotide calls made using the CaVEMan and indels called by Pindel alone were retained in accordance with the reporting of genomic data.^9^ Clinical characteristics of the patients were picked up from the Supplementary Material in the publications.

### Statistical analyses

For each gene, a Fisher exact test was used to assess differences in the population frequencies of mutations (SNV and indels) in this cohort, versus premenopausal, non-Asian breast cancer subjects from TCGA for Fig. 2, and versus TCGA + ICGC combined for Fig. 3. The Benjamini-Hochberg method of computing the False Discovery Rate was used to adjust for multiple hypothesis testing.

OS distributions were estimated using the Kaplan–Meier method; differences in survival from initial diagnosis were assessed with log-rank tests. Adjusting for age at diagnosis and breast cancer subtype, two multivariable Cox proportional hazards regression models (one to assess the associations of genetic alteration status with OS, another to assess the associations of pathway alterations with OS) were estimated. For the model assessing genetic alteration associations, variable selection was performed via best subsets selection using the Akaike Information Criterion, constrained with the compulsory inclusion of age and subtype. Thirteen of the most commonly altered genes (alteration frequency of 13% and above) were selected for this multivariable analysis of single gene alterations. For the model assessing pathway alteration associations, due to high pairwise correlations among pathway alterations and issues of multicollinearity, variable selection was performed via regularized coefficient shrinkage using the LASSO (Least Absolute Shrinkage and Selection Operator) technique, leaving age, and subtype unpenalized.

### Availability of data

The datasets generated and/or analyzed are available as supplementary files. Additional sequence data have been deposited in the NCBI Short Read Archive https://www.ncbi.nlm/gov/sra, with accession number SRP150940.

## Electronic supplementary material

Supplementary TablesConsent Form
